# Proteomic and metabolomic profiles of plasma-derived Extracellular Vesicles differentiate melanoma patients from healthy controls

**DOI:** 10.1016/j.tranon.2024.102152

**Published:** 2024-10-13

**Authors:** SM Bollard, J Howard, C Casalou, BS Kelly, K O'Donnell, G Fenn, J O'Reilly, R Milling, M Shields, M Wilson, A Ajaykumar, K Triana, K Wynne, DJ Tobin, PA Kelly, A McCann, SM Potter

**Affiliations:** a Department of Plastic & Reconstructive Surgery, Mater Misericordiae University Hospital, Dublin 7, Ireland; b School of Medicine, University College Dublin, Dublin 4, Ireland; c Conway Institute of Biomolecular and Biomedical Research, University College Dublin, Dublin 4, Ireland; d Charles Institute of Dermatology, University College Dublin, Dublin 4, Ireland; e UCD-Clinical Research Centre, University College Dublin, Dublin 4, Ireland; f Systems Biology Ireland, University College Dublin, Dublin 4, Ireland; g UCD School of Veterinary Medicine, University College Dublin, Dublin 4, Ireland

**Keywords:** Melanoma, Extracellular Vesicle, Biomarkers, Proteomics, Metabolomics

## Abstract

**Background:**

Plasma-derived Extracellular Vesicles (EVs) have been suggested as novel biomarkers in melanoma, due to their ability to reflect the cell of origin and ease of collection. This study aimed to identify novel EV biomarkers that can discriminate between disease stages. This was achieved by characterising the plasma-derived EVs of patients with melanoma, and comparing their proteomic and metabolomic profile to those from healthy controls.

**Methods:**

EVs were isolated from the plasma of 36 patients with melanoma and 13 healthy controls using Size Exclusion Chromatography. Proteomic and Metabolomic Analyses were performed, and machine learning algorithms were used to identify potential proteins and metabolites to differentiate the plasma-derived EVs from melanoma patients of different disease stages.

**Results:**

The concentration and size of the EV population isolated was similar between groups. Proteins (APOC4, PRG4, PLG, TNC, VWF and SERPIND1) and metabolites (lyso PC a C18:2, PC ae C44:3) previously associated with melanoma pathogenesis were identified as relevant in differentiating between disease stages.

**Conclusion:**

The results further support the continued investigation of circulating plasma-derived EVs as biomarkers in melanoma. Furthermore, the potential of combined proteo-metabolomic signatures for differentiation between disease stages may provide valuable insights into early detection, prognosis, and personalised treatment strategies.

## Highlights

• Plasma-derived Extracellular Vesicles (EVs) from patients with melanoma have similar size and concentration compared to those from healthy controls.
• Proteins linked to melanoma development (PRG4, APOC4, SERPIND1, VWF, TNC and PLG) were identified in the plasma-derived EVs of patients with melanoma.
• Metabolomic analysis revealed alterations in phosphatidylcholines in the plasma-derived EVs of patients with melanoma, although their definitive role is unclear.

## Introduction

The successful treatment of patients with melanoma remains a significant challenge due to the paucity of reliable biomarkers for prognostication and monitoring of disease progression. Risk stratification of patients with melanoma in clinical practice primarily relies on Breslow Thickness (BT). However, the use of BT as a prognostic indicator remains unsatisfactory, as those with a BT of <1 mm, which carries a 95 % 5-year survival rate [1], still account for close to a third of melanoma related deaths [2,3]. Thus, there is a pressing need for improved biomarkers in melanoma to enhance prognostication and enable early detection of disease recurrence.

Extracellular Vesicles (EVs) have been suggested as a novel source of biomarkers in melanoma as well as other malignancies. They have been suggested as a potential “liquid biopsy” source, due to their ability to reflect the contents of the cell from which they have been derived [4], and their identification in multiple different bodily fluids [[5], [6], [7], [8], [9], [10]]. They carry a diverse range of cargo, including proteins, lipids, metabolites, RNA, and DNA. Proteomic analyses, involving the unbiased large-scale analysis of the entire protein content of a sample, have long been established in biomarker discovery. However, in recent years, metabolomics, involving the comprehensive profiling of all metabolites in a sample, has garnered increasing interest in the cancer biomarker community [11]. Moreover, the integration of different "omic" analyses through "multi-omic" approaches has gained popularity [12], allowing for a more comprehensive understanding of complex biological processes. These combined techniques generate vast amounts of data, and with the advent of advanced bioinformatics and machine learning, as they facilitate the identification of molecules and targets of interest that can serve as biomarkers. This work aimed to describe the variations in plasma-derived EV cargo across melanoma stages, as a basis for future longitudinal biomarker studies.

## Materials and methods

### Participant recruitment

Participants were recruited from a tertiary university hospital following Institutional Review Board approval (Ref: 1/378/2189). Patients diagnosed with melanoma undergoing surgical treatment or attending the clinic were eligible for recruitment. The inclusion criteria for the study were as follows: fluency in written and spoken English, capability of performing written informed consent and an age of 18 years or older. Participants were recruited to one of three groups: Group 1 (Primary Melanoma): those with a new histological diagnosis of melanoma within 30 days of surgical excision, Group 2 (Metastatic Melanoma) those within 30 days of histological, clinical, or radiological diagnosis of recurrence/progression, or Group 3 (Healthy Control) those with no history of malignancy attending clinic for unrelated reasons. Participants were excluded if they had given a blood donation >450 mL within 30 days. Clinical data were gathered, and blood was collected in an EDTA tube through direct venepuncture. Samples were spun at 3000 x *g* for 10 min in line with local clinical protocols, before plasma was aliquoted and stored at −80 °C. Upon thawing, samples were centrifuged again at 2500 x *g* for 15 min to obtain platelet poor plasma.

### Extracellular Vesicle isolation

Extracellular Vesicles (EVs) were isolated from the plasma using Size Exclusion Chromatography (SEC) using iZon qEVoriginal Gen2 70 nm columns (iZon Science, #ICO-70). For each isolation, the column was flushed with 17 mL of freshly filtered PBS, and 500µL of plasma was added to the loading frit. A purified collection volume (PCV) of four x 400µL fractions following a buffer volume of 2500µL was collected for downstream analysis.

### Nanoparticle tracking analysis

Particle concentration and size were assessed with Nanoparticle Tracking Analysis (NTA) using a NS3000 with a 488 mm laser. Samples were diluted in fresh filtered PBS (1:25) and loaded using a syringe pump, analysing under constant flow (flow rate = 50) at 25 °C, at camera level 12 and screen gain 2. Five x 60-second videos were captured for each sample and analysed using NTA 4.1 software with a detection threshold of 10 and bin size of 2.

### Western blot

Sodium dodecyl sulphate polyacramide (SDS-PAGE) gel electrophoresis and Western blotting were performed as described previously [13]. The following antibodies were used: Alix (1:1000, Abcam, #ab186728), Calnexin (1:2000, Abcam, #ab112995), CD63 (1:1000, Abcam, #ab271286), Apolipoprotein E (1:1000, Santa Cruz Biotechnology, #sc13538).

### Proteomic analysis

A volume of 400μL was extracted from the plasma-derived EVs, and concentration was achieved using an Amicon 10 kDa centrifugal filter (Millipore, #UFC9010). The solution was then treated with 8 M Urea/50 mM Tris-HCL and sonicated, followed by reduction using 8 mM DTT and carboxylation with 20 mM iodoacetamide. After dilution, overnight digestion with trypsin was performed on the samples, terminated by formic acid, and cleaning was executed using C18 HyperSep™ Spin Tip Microscale SPE Extraction Tips. Dehydration of the resultant pellets was carried out and they were stored at −20 °C. Mass spectrometry was performed on a Bruker timsTof Pro mass spectrometer coupled with an Evosep One Liquid chromatography system. The spectrometer was operated in positive ion mode, utilising Trapped Ion Mobility Spectrometry (TIMS) and Parallel Accumulation Serial Fragmentation. A scan range of 100 – 1700 *m/z* was performed at a rate of 5 PASEF MS/MS frames to 1 MS scan with a cycle time of 1.89 s. The raw data was analysed using MaxQuant (release 2.0.2.0) against the homo sapiens dataset from the Uniprot Swissprot database, employing specific parameters for TIMS DDA for human samples. Peptide scores corresponding to a False Discovery Rate of 0.01 were accepted from the search, and the normalised protein intensity of each identified protein was used for Label Free Quantification. All analyses were performed in duplicate, with each duplicate regarded as an independent quantification for the corresponding study group.

### Metabolomics

Plasma-derived EVs (400μL) were concentrated using an Amicon 10 kDa centrifugal filter. Samples were then dried under a nitrogen stream, resuspended in a mixture of ethanol/PBS, sonicated, and snap-frozen. After two cycles of this process, the samples were centrifuged at 24,000 x *g* for 5 min at 2 °C, and the supernatant was used for metabolomic analysis. The AbsoluteIDQ® p180 assay (Biocrates Life Sciences, Innsbruck, Austria), a targeted metabolomics platform, was utilized for the metabolomic analysis. The supernatant was dried under a stream of nitrogen for 30 min, treated with a 5 % phenyl isothiocyanate derivatisation solution, and the metabolites were extracted with 5 mM ammonium acetate in methanol. The eluate was then diluted for LC-MS/MS run and flow injection analysis-tandem mass spectrometry. The analysis was performed on a SCIEX QTRAP 6500plus mass spectrometer coupled with a SCIEX ExionLC™ Series Ultra-High-Performance Liquid Chromatography. Amino acids and biogenic amines were quantified using isotopically labelled internal standards and 7-point calibration curves, while other metabolites like acylcarnitines, lysophosphatidylcholines, phosphatidylcholines, and sphingomyelins were measured semi-quantitatively using 14 internal standards, all processed within the MetIDQ™ software. Analyses were conducted in duplicate, and the obtained concentrations were subsequently averaged to yield a single representative concentration for each sample.

### Data analysis

Proteomic and metabolomic data, as obtained from MaxQuant (release 2.0.2.0) and MeltIDQ™ respectively as described, were subsequently analysed using R (v4.2.3). Proteins or metabolites missing >40 % of values were removed from the datasets, and remaining missing values were imputed using a random forest algorithm [14]. From the proteomic dataset, immunoglobulins were also removed as in other similar studies within the literature, as these are abundant serum and plasma proteins which can mask differences in low abundance proteins of interest [15,16]. Data were then normalised and log-transformed. After the datasets had been analysed individually, they were combined in a ‘multi-omics’ approach to develop a proteo-metabolomic signature. Comparison with the ExoCarta top 100 EV proteins [17], STRING analysis, differential expression analysis and machine-learning based feature selection were conducted. A p-value of <0.05 was defined as the threshold for significance upon differential expression analysis. Feature selection was performed using k-Nearest Neighbour (kNN), logistic regression with Least Absolute Shrinkage and Selection Operator (LASSO) regularisation, and random forest model. This yielded feature sets for each model, and also a subset of features that were identified by all models. For each feature set unsupervised hierarchical clustering according to Euclidean distance was performed. Performance of this clustering for classification between study groups was assessed. Principal Component Analysis (PCA) was used for visualisation of the feature space.

The R packages used for these analyses included readxl (v1.4.2), Biobase (v2.58.0), MissForest (v1.5), limma (v3.54.2), class (v7.3–21), pROC (v1.18.0), caret (v6.0–94), glmnet (v4.1–7), randomForest (v4.7–1.1), dynamicTreeCut (v1.63–1), aricode (v1.0.2), ggplot2 (v3.4.1), ggfortify (v0.4.16), reshape2 (v1.4.4) and pheatmap (v1.0.12).

EV characterisation, proteomic and metabolomic data were analysed using GraphPad Prism (v9.5.1) for generating ROC-AUC curves and comparing group variables. The Shapiro-Wilk test checked variable distributions, and Welch's *t*-test, One-way ANOVA, Mann-Whitney U or Kruskall-Wallis tests were used for hypothesis testing as necessary. Correlations were assessed using Spearman's correlation. Chi-squared tests compared categorical variable distributions. In specific cases, Monte Carlo simulations were performed. Data were represented as mean ± standard deviation or median [IQR] as required, with *p*-values under 0.05 indicating significance.

## Results

### Participant demographics

Study participant demographics and clinicopathological characteristics are summarised in Table 1. A total of 36 patients with melanoma and 13 healthy controls were recruited to the study over the study period (Jan 2021 – Dec 2022). The patients with melanoma included 24 patients with a new diagnosis of melanoma (primary melanoma group) and 12 with a new diagnosis of disease progression (metastatic melanoma group). Age varied significantly between the three study groups of primary melanoma, metastatic melanoma and healthy controls (*F (2,46) = 2.52, p* *<* *0.001, One-way ANOVA)*. Post-hoc comparisons using Tukey's test showed that the primary melanoma and metastatic melanoma groups were similar to each other in relation to age, but both groups were significantly older when compared to healthy control group (*p < 0.001*). Data to calculate the Charlson Comorbidity Index (CCI) was available for *n =* 23 patients in the primary group and *n =* 9 in the metastatic group. The metastatic group had a significantly higher median CCI compared to the primary group, with median scores of 8 [[2], [3], [4], [5], [6], [7], [8], [9], [10]] and 4 [[2], [3], [4], [5], [6]] respectively (*W = 71.5, p = 0.023, Mann-Whitney U test*). There was no significant difference in sex, ASA classification or smoking status between the groups.

**Table 1:** Participant demographics and clinicopathological characteristics.

	Primary Melanoma (*n* = 24)	Metastatic Melanoma *n* = 12)	Healthy Controls (*n* = 13)		
Age, years *(mean ± SD)*					
Age, years *(mean ± SD)*	62.67 ± 15.66	62.42 ± 22.48	34.76 ± 14.07	*F(2,46) = 12.52*	*p* < 0.001^†^
**Sex (*n*)**					
Male	16	7	4	*Χ^2^(2) = 4.46*	*p* = 0.108‡
Female	8	5	9		
**American Society of Anaesthesiologists Physical Status Classification (*n*)**					
I	8	3	–	*maxT = 0.74*	*p* = 0.881^•^
II	9	5	–		
III	5	2	–		
IV	–	–	–		
Unknown	2	2	–		
**Charlson Comorbidity Index (CCI)**					
CCI (*median [IQR])*	4 [[2], [3], [4], [5], [6]]	8 [[2], [3], [4], [5], [6], [7], [8], [9], [10]]	–	*W = 71.5*	p = 0.023*
Unknown	1	3	–		
**Smoking Status (*n*)**					
Smoker	1	1	–	*maxT = 1.26*	*p* = 0.660^•^
Ex-Smoker	11	3	–		
Non-Smoker	11	6	–		
Unknown	1	2	–		
**Breslow Thickness (BT)**					
BT, mm (*median [IQR]*)	0.85 [0.56 – 1.13]	3.50 [2.00 – 5.45]	–	*W = 235.5*	*p* = 0.002^†^
**Subtype**					
Superficial Spreading (SSMM)	16	5	–	*maxT = 2.81*	*p* = 0.019^•^
Nodular Melanoma	1	5	–		
Lentigo Maligna Melanoma (LMM)	7	–	–		
Acral Lentiginous	–	1	–		
Unknown	–	1	–		
**Melanoma Site**					
Head & Neck	8	1	–	*maxT = 1.61*	*p* = 0.394^•^
Upper Limb	5	3	–		
Trunk	5	3	–		
Lower Limb	6	5	–		
**Primary Tumour Ulceration Present**					
Yes	4	5	–	*maxT = 2.52*	*p* = 0.021^•^
No	20	5	–		
Unknown	–	2	–		

P values were determined with ^†^ one-way ANOVA (F)

‡ Pearson's Chi-squared test (Χ^2^), Chi-squared test with Monte Carlo simulation (maxT), and * Mann Whitney U test (W).

The median BT differed significantly between primary and metastatic melanoma groups (*W = 235.5,p = 0.002, Mann-Whitney U test*). Specifically, the primary melanoma group had a median BT of 0.85 [0.56 – 1.13] mm, which was significantly lower than the median BT of 3.50 [2.00 – 5.45] mm in the metastatic melanoma group. There were also significant differences in the distribution of the melanoma subtypes between groups (*maxT = 2.81, p = 0.019, Chi-squared test with Monte Carlo simulation*), with a higher proportion of Nodular Melanoma seen in the metastatic melanoma group, and a higher proportion of Lentigo Maligna Melanoma (LMM) seen in the primary melanoma group. There was no significant difference in the distribution of melanoma site between groups (*maxT = 1.61, p = 0.394, Chi-squared test with Monte Carlo simulation*). There was a higher proportion of ulcerated tumours seen in the metastatic melanoma group compared to the primary melanoma group (*maxT = 2.52, p = 0.021, Chi-squared test with Monte Carlo simulation*).

### Extracellular Vesicle characterisation

**Fig. 1:**
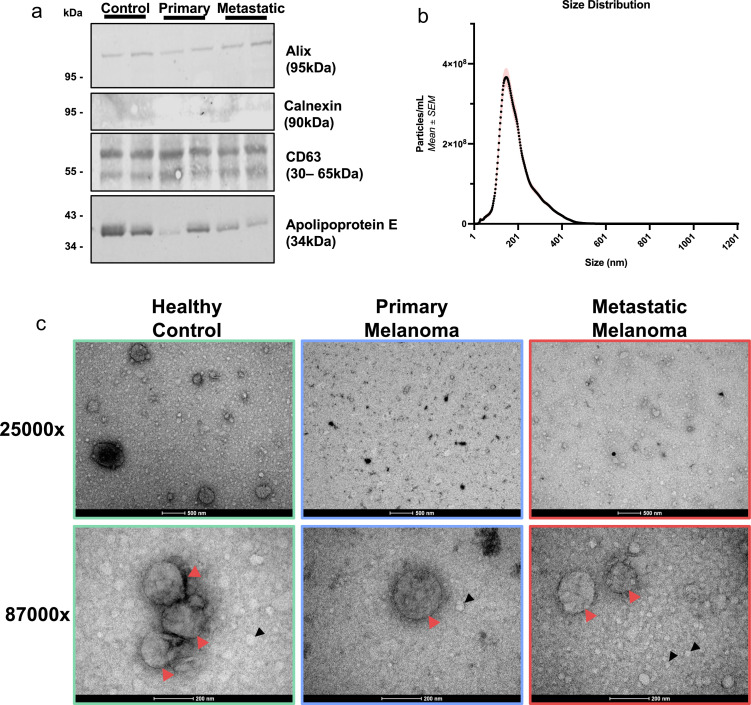
**Size Exclusion Chromatography isolated EVs from patients with primary and metastatic melanoma, and healthy controls demonstrate characteristic EV markers, size distribution and morphology**. A) Western Blot analysis showing relevant EVs characterisation markers (MISEV2023), showing the presence of CD63 and Alix, and absence of Calnexin in the plasma-derived EVs isolated from representative samples from all three study groups. Apolipoprotein E (APOE), a known co-isolates of plasma EVs isolated using SEC, are observed in all groups. B) Size distribution curve (NTA) for all samples is seen here, demonstrating a peak at ∼200 nm indicating successful enrichment for small EVs C) TEM images taken at 25000x and 87000x, showing the expected cup-shaped morphology of the EVs (red arrows). Lipoproteins (black arrows) can be observed in all groups.

Representative samples were selected from each of the three study groups (Primary Melanoma, Metastatic Melanoma & Healthy Controls), and plasma-derived EVs were isolated and concentrated using centrifugal ultrafiltration. Western Blot analysis (Fig. 1A) was used to confirm the presence of CD63 and Alix in the plasma-derived EVs isolated from all three study groups. Calnexin was not detected in the samples indicating that isolated EVs do not originate from the Endoplasmic Reticulum (ER). A limited amount of contamination was detected by the presence of Apolipoproteins in all samples, recognised as co-isolated structures in plasma-derived EV isolation. The size of the particles was analysed using NTA (Fig. 1B). The size distribution curve of all samples demonstrated a peak at approximately 200 nm, indicating that the PCV was enriched for small EVs (<200 nm) as expected. TEM imaging confirmed the presence of cup shaped EVs within the expected size range in all groups, as well as the co-isolation of apolipoproteins (Fig. 1C).

### Plasma-derived EV comparison of concentration, protein contamination and size distribution among study groups

**Fig. 2:**
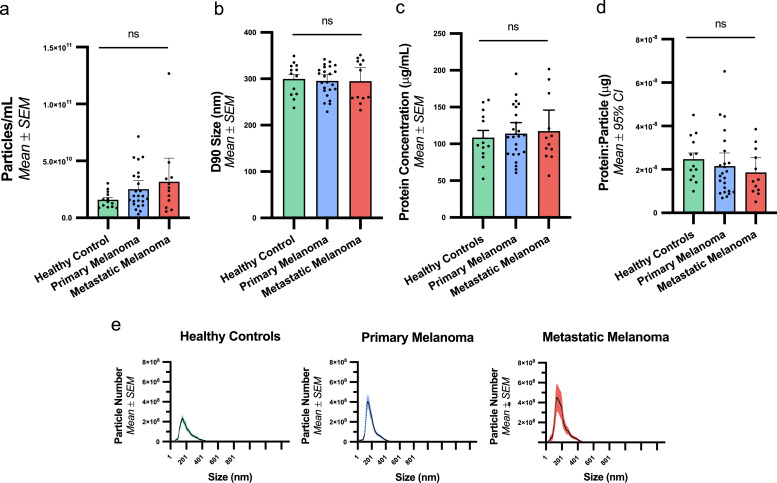
**Comparison of plasma-derived EV characteristics observed among patients with primary melanoma, metastatic melanoma, and healthy controls**. Analysis using NTA showed A) no significant difference in the plasma EV concentration (*H(2) = 2.80, p = 0.247, Kruskal-Wallis test*) or B) the D90 (the size at which 90 % of particles are below) between groups (*H(2) = 0.25, p = 0.884, Kruskal-Wallis test*). Analysis of C) protein concentration (μg) *(F(2, 46) = 0.1675, p = 0.846, One-way ANOVA*) and D) protein:particle (μg) ratio (*H(2) = 2.41, p = 0.299, Kruskal-Wallis test*) using the BCA Protein assay also showed no significant difference between the groups. E) All three groups had a comparable EV size distribution profile.

The characteristic properties of plasma-derived EVs from patients with primary melanoma, metastatic melanoma, and healthy controls were compared using both NTA to analyse EVs size and concentration, and BCA for protein quantification as summarised in Fig. 2. There was no significant difference in the concentration of plasma-derived EVs between groups (Fig. 2A, (*H(2) = 2.80, p = 0.247, Kruskal-Wallis test*). Similarly, there was no difference in the size of the plasma-derived EVs between groups. This was evidenced by the modal size (*F(2, 46) = 0.66, p = 0.521*), and the D90 analysis, which describes the size (nm) at which 90 % of particles are below (*H(2) = 0.25, p = 0.884, Kruskal-Wallis test,* Fig. 2B). Furthermore, the size distribution curves of the EVs isolated from the plasma of each study group appeared similar (Fig. 2E). Analysis of protein concentration of the EV elute and the protein to particle ratio, used as a measure of protein content per particle in this instance as opposed to its inverse ratio used for purity measurement described above, also showed no significant difference *(protein concentration F(2, 46) = 0.17, p = 0.846, One-way ANOVA; protein:particle ratio H(2) = 2.41, p = 0.299, Kruskal-Wallis test*; Fig. 2C and D).

### Proteomic and metabolomic analysis overview

Two outlier samples from the primary melanoma group were removed due to a unique metabolomic profile in a pregnant patient, and suspected processing anomalies in a single sample. MaxQuant identified 257 proteins across all remaining samples, which was reduced to 222 after excluding unreliable identifications. Of these 222 proteins, 87 were involved in the vesicle-mediated transport pathway (GO:0,016,192), with 14 among ExoCarta's top 100 EV proteins [17]. All proteins were detected in melanoma patient samples, two of which were exclusive to these patients (HIST1H1E, ANKHD1). A total of 88 metabolites were identified, with each metabolite identified in all three study groups. The majority of identified metabolites were glycerophospholipids (72/88) and sphingolipids (14/88), key constituents of cellular membranes, as well as one biogenic amine (Spermidine) and one amino acid (Serine). Following data pre-processing as described in the methods, 107 proteins and 86 metabolites were included in the complete dataset for further analysis.

### Protein and metabolite signatures distinguish plasma-derived EVs of patients with melanoma from healthy controls

**Fig. 3:**
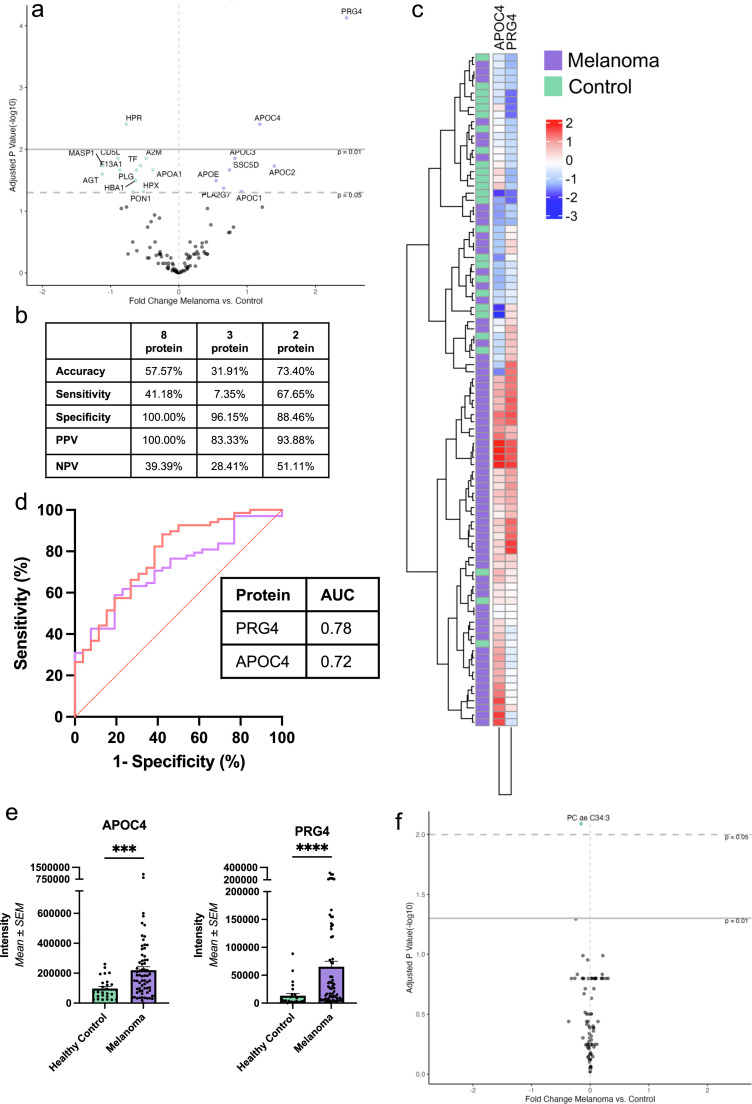
**Plasma-derived EV proteomic and metabolomic analysis of patient with melanoma and healthy controls reveals differential expression and clustering patterns** A) Volcano plot showing variations in protein expression in melanoma patients' plasma-derived EVs versus those from healthy controls. Proteins significantly more abundant in melanoma samples are marked in purple, those less abundant are in green. B) Performance metrics of the 8 protein signature from kNN, 3 common proteins across all algorithms, and the 2-protein multi-algorithm signature. C) Unsupervised hierarchical clustering, using the multi-algorithm protein signature (APOC4, PRG4), segregates samples with 73.4 % accuracy. D) ROC curves for PRG and APOC4. E) APOC4 and PRG4 are significantly more expressed in melanoma patient-derived EVs relative to healthy controls (PRG*: p < 0.001,* APOC4*: p < 0.001, Mann-Whitney U test*). F) Volcano plot showing differential abundances of metabolites in plasma-derived EVs of patients with melanoma in comparison to healthy controls. A single significantly decreased metabolite (PC ae C34:4) in EVs derived from melanoma patients is represented in green.

Proteomic, metabolomic, and proteo-metabolomic analyses revealed distinctive plasma-derived EV signatures among patients with melanoma in comparison to healthy controls. Differential expression of circulating plasma-derived EV proteins in the proteomic analysis highlighted 20 significant proteins that were differentially abundant between patients with melanoma and controls (Supplementary Table 1). Specifically, 8 proteins were increased while 12 were decreased in the plasma-derived EVs of patients with melanoma (Fig. 3A). Serial k-nearest neighbour (kNN) algorithms identified a signature consisting of eight proteins at *k =* 1, which differentiated melanoma patients from controls with an accuracy of 57.45 % (46.82 – 67.60 %), which was not significantly different from the “No Information Rate” (NIR) of 72.34 % (*Accuracy (ACC) > NIR, p = 0.999*). This signature was then refined using a multi-algorithm approach (Fig. 3B). A logistic regression with LASSO penalty model selected 28 proteins relevant to classification, and the top 10 % most important features were identified also using a random forest. Three proteins were commonly identified using the three algorithms as relevant in classifying EVs derived from patients with a melanoma diagnosis compared to those from healthy controls: specifically, Proteoglycan 4 (PRG4), Apolipoprotein C4 (APOC4) and Haptoglobin-Related Protein (HPR). A grid search demonstrated that APOC4 and PRG4 could be used to distinguish melanoma patient EVs from healthy control with an accuracy of 73.4 % (Fig. 3C). However, this was also not significantly different to the NIR (*Acc > NIR, p = 0.461*). A ROC-AUC analysis of these two markers was performed, using the raw proteomic data, and both APOC4 and PRG4 were found to have satisfactory AUCs (Fig. 3D), and both displayed significantly higher intensities in the melanoma patient EVs in comparison to controls (PRG4*: W = 390, p < 0.001;* APOC4*: W = 498, p < 0.001, Mann-Whitney U test*; Fig. 3E). Importantly, the PRG4 intensity demonstrated a weakly positive significant correlation with the age (years) of the participants in the entire cohort, though there was no significant correlation between age (years) and APOC4 intensities *(PRG4: r = 0.34, p = 0.021; APOC4: r = 0.087, p = 0.660, Spearman's correlation).*

Metabolomic analysis of plasma-derived EVs revealed varying metabolite levels between the patient and control groups, with PC ae C34:3 significantly decreased in melanoma patients *(log2 fold change = −0.16, p = 0.008*; Fig. 3F). The one significantly decreased metabolite (PC ae C34:3) was selected as the optimal feature to differentiate between the groups by the serial kNN models, with a local maximum AUC point of 0.76 at *k* = 8 and *N* = 1. A more lenient selection criterion was applied to identify potential metabolites, with logistic regression with LASSO penalty and random forest model pinpointing two common metabolites: PC ae C34:3 and lysoPC a C18:2. Together, they achieved a classification accuracy of 82.98 % and sensitivity of 85.29 %, albeit not significantly superior to the NIR (*Acc > NIR, p = 0.*066). However, there was no significant difference in the concentration between the two groups upon direct comparison (PC ae C34:3*: W = 219, p* *=* *0.968;* lysoPC a C18:2*: W = 201, p = 0.642, Mann-Whitney U test*).

**Fig. 4:**
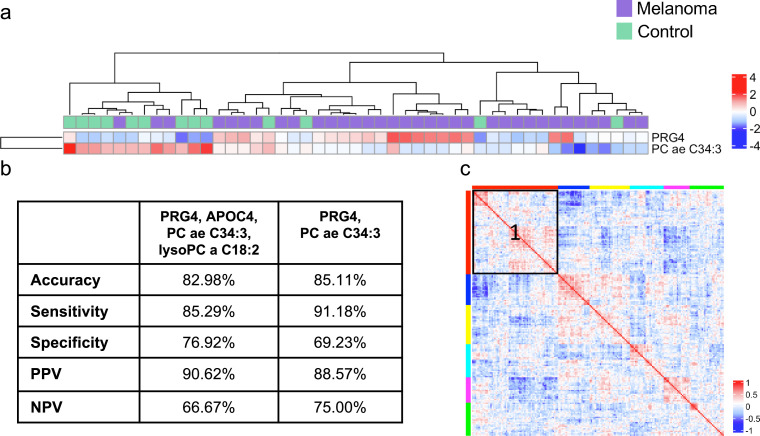
**Proteo-metabolomic combined signature improves classification of plasma-derived EVs from patients with melanoma from healthy controls** A) Unsupervised hierarchical clustering of samples differentiating plasma-derived EV samples of patients with melanoma from healthy controls, based upon the multi-algorithm proteo-metabolomic signature of PRG4 and PC ae C34:4. B) Performance metrics of the combined protein and metabolomic features distinguishing plasma-derived EVs of patients with melanoma from healthy controls. The combination of all four proteins and metabolites performed with a similar accuracy as the multi-algorithm metabolomic signature alone. Performance improved upon selecting best performing features using a grid search, resulting in a signature of one protein (PRG4) and one metabolite PC ae C34:3. C) Similarity Matrix based upon Spearman's correlation, clustered into 6 partitions. Both PRG4 and PC ae C34:3 are within the cluster marked 1 (red).

To further improve the classification accuracy and to develop a proteo-metabolomic signature, the datasets were combined in a ‘multi-omics’ approach. The clustering ability of the four previously identified proteins and two metabolites were assessed. The combined model achieved an accuracy of 82.99 % (Fig. 4A), which was identical to the metabolomics alone and was also not significantly better than the NIR (*p* = 0.066). A grid search was then performed, and it was found that the best performing combination of features in terms of accuracy to distinguish the plasma-derived EV samples from patients with melanoma compared to healthy controls, was a signature consisting of PRG4 and PC ae C34:3 (Fig. 4B). This combination achieved 85.11 % accuracy and was significantly better than the NIR (*p* = 0.031). Both PRG4 and PC ae C34:4 were also identified within the same cluster of Spearman's similarity matrix, suggesting their expression may be correlated (Fig. 4C).

### Protein and metabolite signatures distinguish plasma-derived EVs of patients with metastatic disease from those with primary melanoma

**Fig. 5:**
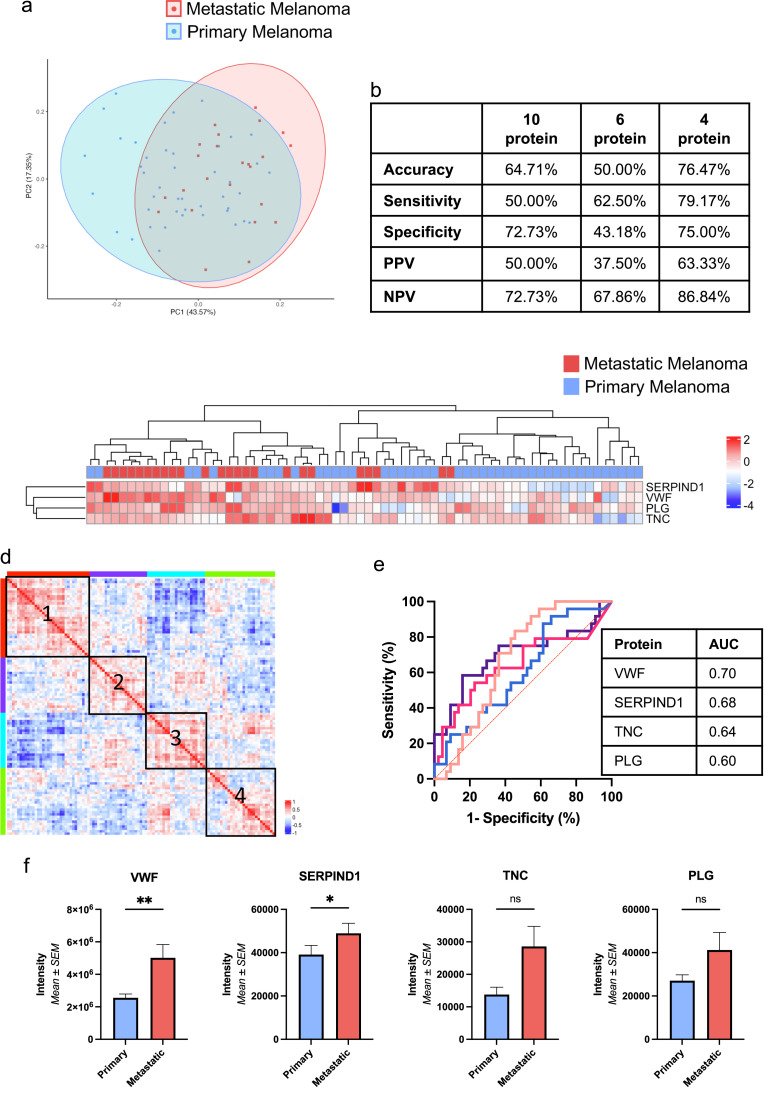
**Protein expression and cluster analysis of plasma-derived EV samples distinguishes patients with metastatic and primary melanoma A)** PCA analysis of the plasma-derived EV samples from patients with metastatic and primary melanoma, using the expression levels of the top 5 significantly differentially expressed proteins, as identified by kNN algorithm (*k = 1*). **B)** Performance metrics of the classification ability of three different protein signatures; 10 proteins identified by kNN, 6 proteins common to all algorithms, and the optimal 4 protein multi-algorithm signature identified using a grid search, displaying superior accuracy. **C)** Heatmap demonstrating unsupervised hierarchical clustering based upon the 4 protein multi-algorithm protein signature, clustering plasma-derived EV samples from patients with metastatic and primary melanoma with an accuracy of 76.47 % (*Acc > NIR, p-value = 0.026*). **D)** Pairwise similarity matrix based upon Spearman's correlation of the proteins identified in the metastatic cohort, clustered into 4 groups. The protein VWF was included in cluster 1 (red), SERPIND1 and TNC in cluster 2 (purple), and PLG in cluster 3 (blue). **E)** ROC analysis of the 4 proteins identified in the multi-algorithm signature. **F)** VWF and SERPIND1 were significantly increased in the plasma-derived EV samples from patients with metastatic disease relative to EVs derived from primary melanoma patients, upon analysis of raw intensity data (VWF*: p = 0.007,* SERPIND1*: p = 0.017, Mann-Whitney test*).

This analysis pathway was repeated to compare the plasma-derived EVs from patients with metastatic and primary melanoma. The top 5 significant differentially abundant proteins as determined by kNN (*k* = 1), were able to distinguish the cohorts upon PCA analysis, though with substantial overlap (Fig. 5A). Performance metrics of a 10-protein kNN signature, a 6-protein signature of proteins common to all algorithms, and the optimal multi-algorithm 4-protein signature are presented in Fig. 5B. The multi-algorithm approach for feature selection resulted in the selection of a 4-protein signature, which could discriminate the plasma-derived EVs of patients with metastatic disease from those with primary melanoma, with an accuracy of 76.47 %, and significantly better than the NIR (*Acc > NIR, p = 0.026*; Fig. 5C). This 4-protein signature consisted of Plasminogen (PLG), Von Willebrand Factor (VWF), Serpin Family D Member 1 (SERPIND1) and Tenascin-C (TNC). Two of the proteins (SERPIND1 and TNC) had correlated abundance levels in the metastatic cohort (Fig. 5D). An ROC-AUC analysis of the four proteins showed AUCs ranging from 0.6 – 0.7 for each individual protein (Fig. 5E), and VWF and SERPIND1 were significantly increased in the samples obtained from patients with metastatic disease (VWF*: W = 321.00, p = 0.007,* SERPIND1*: W = 343.00, p = 0.017, Mann-Whitney U test; Fig. 3.8F).* There was no significant difference in the raw intensities of TNC *(W = 377.00, p = 0.053, Mann-Whitney U test*) or PLG *(W = 419.00, p = 0.165, Mann-Whitney U test)*.

Upon analysis of the metabolomic profile of plasma-derived EVs in this cohort, no metabolites were significantly different in the EVs of patients with metastatic melanoma compared to those with primary melanoma. The top 7 most significant metabolites were identified as the optimal cut-off for classification by the kNN algorithm (*k = 1*). The logistic regression with LASSO penalty identified a single metabolite (PC ae C44:3) and a less stringent selection process was adopted to generate a panel prior to the gird search. Finally, a 2-metabolite signature was identified through the multi-algorithm approach, consisting of PC aa C38:0 and PC ae C44:3, which differentiated the cohorts of patients with metastatic and primary disease with 70.59 % accuracy (*Acc > NIR, p = 0.300*). No significant difference was seen in the concentration of the metabolites in the plasma-derived EV samples of those with metastatic compared to those with primary melanoma (PC aa C38:0: *p = 0.249,* PC ae C44:3: *p = 0.922, Mann-Whitney U test)*. The combination of the identified proteomic and metabolomic datasets revealed the optimal features for differentiation were 2-proteins, VWF and SERPIND1 which classified the samples between those with metastatic and primary melanoma with 79.41 % accuracy (*Acc > NIR, p = 0.049*). No metabolites were identified as relevant to the final proteo-metabolomic signature to differentiate the EVs from patients with metastatic disease from those with primary melanoma. The proteins and metabolites identified in all signatures with associated performance metrics are presented in Supplementary Tables 2–7.

## Discussion

This is the first study to analyse the combined proteomic and metabolomic profiles of the plasma-derived EVs from patients with melanoma, discerning EV-derived biomarkers that allow the differentiation of patients with melanoma from healthy controls, and those with primary from metastatic disease. Plasma-derived EVs were isolated using SEC, which has been reported to minimally alter EV preparations and preserve its functionality compared to other techniques [18]. SEC has also been demonstrated to be suitable for downstream proteomic and metabolomic analyses [19]. Importantly, while SEC can enrich for small EVs (<200 nm), as seen in our results, the isolate contains a heterogenous population of particles, as it also includes larger EVs and known co-isolates of SEC, lipoproteins [20]. Whilst other techniques can be used to improve the purity of preparations [20,21], using SEC alone uniformly across all samples still allows for direct comparison of protein and metabolite content between groups, regardless of the population's heterogeneity. Furthermore, SEC alone is a rapid and inexpensive technique, making it a strong candidate for use in biomarker discovery due to its’ potential clinical translatability [22].

The concentration and size of plasma-derived EVs from melanoma patients were similar in size and concentration when compared to healthy controls, or across different melanoma disease stages. These findings align with previous studies by Peinado et al. [23] and Cordonnier et al. [24]. Interestingly, this study did not observe the increase in protein concentration per particle with escalating clinical stage reported by Peinado et al. [23]. This discrepancy may be due to different EV isolation techniques utilised, given that different isolation methods isolate distinct EV populations [25,26]. However, due to the presence of lipoproteins among measured particles isolated using SEC, the protein concentration of plasma-derived EVs isolate might not serve as an effective biomarker, necessitating further exploration of additional EV features. Several plasma-derived EV protein markers of melanoma have been previously suggested, for both the purposes of melanoma detection and monitoring of progression due to their alteration with advancing disease stages, including HSP70, CAV1, MCAM, MART-1, TYRP1, TYPR2, ITGA4, CSPG4, MIA, S100B, MET, TNC and PD-L1 [23,24,[27], [28], [29], [30], [31], [32]]. Interestingly, the only protein marker of these identified in this study was TNC, as described by Lattmann et al. (2024), who also used SEC for EV isolation [32]. This supports the understanding in the literature that different EV isolation methods enrich for distinct EV populations [25,26], leading to the identification of different proteins. While this finding is encouraging, it also limits the comparability of proteomic and metabolomic profiles of plasma-derived EVs obtained using different isolation methods. Furthermore, whilst some techniques can enrich for EVs containing tumour- or source-specific markers, it is important to note that the cellular origin of plasma-derived EVs cannot currently be definitively determined, and that these, and other, potential biomarkers may be produced by any cell in response to tumour signals or cancer-related processes.

However, this study has identified other proteins in melanoma patient-derived EVs using machine learning algorithms, including PRG4 and APOC4, that have been linked to melanoma pathogenesis and progression. In the setting of melanoma diagnosis, PRG4 has been identified as one of the most significantly mutated genes in a genome sequencing of 25 metastatic melanoma tumours [33]. PRG4 has also been identified as a gene whose expression can be used distinguish primary melanomas from subcutaneous metastases, in their interrogation of The Cancer Genome Atlas (TCGA) [34]. Similarly, APOC4 was identified as a 200-gene signature for melanoma diagnosis, in a study assessing the relevance of genes across multiple genetic microarray datasets [35].

Similarly, the proteins identified here as potential biomarkers for differentiation of EVs from primary and metastatic melanoma patients have also been implicated in melanoma progression. SERPIND1 has been identified in the circulating serum-derived EVs of patients with a prior history of melanoma [36]. VWF is not only a mediator of platelet adhesion but has also recently been described as a mediator of metastasis in melanoma, as well as other malignancies [37,38]. Downregulation of PLG has been implicated in the success of treatments, with its upregulation associated with a shortened patient survival [39]. TNC is secreted by both melanoma cells and fibroblasts. Its expression increases with the transformation of melanocytes into melanoma cells [40], and it has been shown to promote melanoma migration and invasion through the dermis, supporting survival of disseminated melanoma cells [41,42]. TNC has also been detected at higher levels in the serum of patients with advanced disease in comparison to those with primary melanoma [43], and in the plasma-derived EVs of melanoma patients [32]. The consistency of the results reported herein with existing literature supports the hypothesis that these proteins could be potential novel EV-based biomarkers for melanoma.

Similar to the proteomic profile, the findings reported in this study present novel perspectives of the EV metabolome in melanoma. Adding to the relatively scarce existing literature of EV metabolomics in melanoma, the results presented here did not identify any metabolites identified in previously published studies [44,45]. The identified metabolites of interest in this study were phosphatidylcholines (PCs) and a lysophosphatidylcholine (lysoPC). When comparing metastatic melanoma and primary melanoma using normalised log transformed data, an increase in PC aa C38:0 and a slight decrease in PC ae C44:3 were observed in the plasma EVs from patients with metastatic melanoma. In comparison between melanoma and healthy control, a mild decrease in PC ae C34:3 and lysoPC a C18:2 was found in the plasma EVs from patients with melanoma. There have been previous reports that these metabolites are related to cancer progression [[46], [47], [48]]. The metabolomic signatures identified here suggest a downregulation of glycerophospholipids (PC aa and PC ae), which is associated with the increased activity of phospholipase A2, known to be related to invasive tumours in breast cancer [47]. Moreover, lysoPC a C18:2 and PC aa C38:0 have been implicated in mediating the obesity-cancer relationship [46]. In addition, in a melanoma-bearing Liebechov minipig model, PC aa C38:0 has been recognised to increase in the plasma during disease progression, and decrease when the melanoma is regressing [48]. However, although four different metabolites were identified as potential differentiating factors between groups using the multi-algorithmic approach, none were significant upon interrogation of the raw data, and as such it is challenging to draw any firm conclusions or infer any defined relevance of the metabolites alone.

The approach of combining proteo-metabolomic features greatly improved the classification of patients with melanoma from healthy controls. Notably, this is the first published report of an integrated proteomic and metabolomic profiling of plasma-derived EVs from melanoma patients. When incorporating both proteins and metabolites for differentiation, the accuracy of classification of patients with melanoma from healthy controls improved to 85.11 %. In this context, PRG4 and PC ae C34:3 did not appear correlated. Although further validation is necessary, these support the potential of integrating proteomics and metabolomics for future EV-based melanoma biomarker discovery.

It is also important to consider the demographics of the study cohort. A definite limitation of this study is that the healthy control group was significantly younger than the melanoma patients, a factor that reflects the practical challenges in recruiting age-matched healthy controls in a clinical setting, particularly when the median age at diagnosis for melanoma is between 60 and 64 years [49]. However, this is particularly relevant as it has been demonstrated that the proteins associated with plasma EVs can change with age [50]. Similarly, there are alternative age-associated diseases which also may result in changes in abundance of the potential protein biomarkers identified here. For example, APOC4 has been detected at higher level in the High-Density Lipids isolated from plasma of patients with coronary artery disease compared to controls. This detection was achieved using Ultracentrifugation, a methodology often used in EV related studies [361]. In addition, circulating concentrations of PRG4 have been shown to inversely correlate with joint space narrowing, and are predictive of osteoarthritis progression [362], exceptionally relevant due to the weak correlation seen in this study between the intensity of PRG4 in plasma-derived EVs and age of the participants. Finally, elevated alternative SERPINs and TNC have been identified in a plasma signature associated with increasing chronological age [51]. As such, the elevation of these proteins within the EVs of patients with melanoma compared to healthy controls in this study may reflect the demographics of the population more likely to develop melanoma, as opposed to a reflection of the tumour itself.

Certain melanoma subtypes, as well as melanoma tumours of higher BT, are typically associated with more aggressive disease [1,52] and thus are expected to be more prevalent in groups of patients with metastatic melanoma. This was reflected in this cohort, as there was a significantly higher tumour BT in the metastatic melanoma group compared to the primary melanoma group. There were also more instances of Nodular Melanoma and ulcerated tumours in the metastatic melanoma group, while the primary melanoma group had a higher proportion of LMMs. However, to discern if these observed differences have a significant impact on the study findings, further in-depth research and larger study populations are necessary. In addition, one protein detected solely in the plasma-derived EVs of melanoma patients was HIST1H1E, a nuclear protein listed as a negative EV marker in the MISEV2023 guidelines [53]. This implies that the plasma-derived EV preparation may not solely have co-isolated lipoproteins, but also apoptotic bodies and cellular debris. While this does not undermine the clinical relevance of the identified signatures, it emphasises the need for further investigation to accurately determine the exact nature of the EVs or particle population under study. Moreover, in future research, these identified signatures should be correlated with long-term follow-up data to evaluate their viability as biomarkers for disease prognosis or recurrence monitoring.

These findings demonstrate that changes in the protein and metabolite cargo of circulating plasma-derived EVs, isolated using a clinically translatable method, can reflect disease stage in melanoma and serve as a potential biomarker. This is also the first example of an integrated proteomic and metabolomic approach to analyse plasma-derived EVs in this setting. Importantly, this work generates many potential options for further study. Currently there exists no definite way to determine the cellular origin of circulating EVs. To progress their potential as biomarkers, it is essential to develop an understanding of the broader role of these EV-associated proteins and metabolites, and to establish if they have functional role in melanoma progression. As such, there is a need to investigate the links between these circulating EV biomarkers and their tumour of origin, to ascertain whether the observed changes in cargo are related directly to tumour burden and microenvironment, or as a result of other systemic cancer-associated changes experienced by patients. In addition, validation of these results, using alternative techniques to quantify proteins, in larger and more diverse patient populations are crucial for establishing the clinical utility of EVs as biomarkers. It is essential to expand the cohort and ensure age-matched comparisons. This approach will enable the differentiation of extracellular vesicle (EV) cargo associated with melanoma from those linked to other diseases that may affect a population with melanoma. Furthermore, assessing the predictive ability of these biomarkers in differentiating those who are likely to develop metastatic disease will provide crucial insights into their potential role in guiding clinical decision-making. Nonetheless, these results establish a strong foundation for further investigation of circulating plasma-derived EVs as biomarkers in melanoma.

## CRediT authorship contribution statement

**SM Bollard:** Writing – original draft, Project administration, Methodology, Investigation, Funding acquisition, Formal analysis, Data curation, Conceptualization. **J Howard:** Writing – review & editing, Methodology, Conceptualization. **C Casalou:** Writing – review & editing, Methodology. **BS Kelly:** Writing – review & editing, Methodology, Conceptualization. **K O'Donnell:** Resources, Project administration, Data curation. **G Fenn:** Writing – review & editing, Data curation. **J O'Reilly:** Writing – review & editing, Data curation. **R Milling:** Writing – review & editing, Data curation. **M Shields:** Writing – review & editing, Data curation. **M Wilson:** Writing – review & editing, Data curation. **A Ajaykumar:** Investigation, Data curation. **K Triana:** Investigation, Data curation. **K Wynne:** Methodology, Investigation, Data curation. **DJ Tobin:** Writing – review & editing, Methodology. **PA Kelly:** Writing – review & editing, Methodology. **A McCann:** Writing – review & editing, Methodology, Conceptualization. **SM Potter:** Writing – original draft, Methodology, Investigation, Funding acquisition, Conceptualization.

## Declaration of competing interest

The authors declare that they have no known competing financial interests or personal relationships that could have appeared to influence the work reported in this paper.

## Appendix Supplementary materials

Image, application 1
