# Supplementary material for: Proteomic and metabolomic profiles of plasma-derived Extracellular Vesicles differentiate melanoma patients from healthy controls
Source: Transl Oncol. 2024 Oct 13;50:102152. doi: 10.1016/j.tranon.2024.102152 (PMC11736400; doi:10.1016/j.tranon.2024.102152)
Supplement: Supplementary file 1 [file mmc1.docx]

**Supplementary Table 1: Significant differential protein abundance in plasma-derived EVs: patients with melanoma vs. healthy controls**

| **Gene Symbol** | **Gene Name** | **Log2 Fold Change** | **Adjusted *P*-Value** |
| --- | --- | --- | --- |
| PRG4 | Proteoglycan 4 | 2.46 | <0.001 |
| APOC2 | Apolipoprotein C2 | 1.40 | 0.019 |
| APOC4 | Apolipoprotein C4 | 1.19 | 0.004 |
| APOC1 | Apolipoprotein C1 | 0.92 | 0.048 |
| APOC3 | Apolipoprotein C3 | 0.82 | 0.014 |
| SSC5D | Soluble scavenger receptor cysteine-rich domain-containing protein | 0.74 | 0.022 |
| PLA2G7 | Platelet-activating factor acetylhydrolase | 0.65 | 0.043 |
| APOOE | Apolipoprotein E | 0.54 | 0.032 |
| APOA1 | Apolipoprotein A1 | -0.38 | 0.022 |
| A2M | Alpha-2-macroglobulin | -0.48 | 0.014 |
| HPX | Hemopexin | -0.52 | 0.048 |
| TF | Serotransferrin | -0.56 | 0.018 |
| PLG | Plasminogen | -0.62 | 0.022 |
| HBA1 | Haemoglobin subunit alpha | -0.63 | 0.032 |
| PON1 | Serum paraoxonase/arylesterase 1 | -0.67 | 0.049 |
| HPR | Haptoglobin-related protein | -0.77 | 0.004 |
| F13A1 | Coagulation factor XIII A chain | -0.87 | 0.022 |
| CD5L | CD5 antigen-like | -0.89 | 0.014 |
| MASP1 | Mannan-binding lectin serine protease 1 | -1.11 | 0.018 |
| AGT | Angiotensin | -1.12 | 0.025 |

**Supplementary Table 2: Performance metrics of proteomic signatures developed by different algorithms to differentiate plasma-derived EVs from patients with melanoma vs. healthy controls**

|  | **KNN Features** | **LASSO** | **Random Forest** | **Proteins from all models** | **Multi-Algorithm Protein Signature** |
| --- | --- | --- | --- | --- | --- |
| **Number of Proteins** | 8 | 28 | 11 | 3 | 2 |
| **Proteins** | APOC4, PRG4, APOC3, A2M, TF, MASP1, HPR, CD5L | SSC5D, APOL1, FCN3, F13A1, HPR, APOA2, APOC2, FGG, FN1, GC, HPX, CAT, A1BG, APOD, SERPIND1, S100A9, CLU, PON1, AFM, MASP1, CAMP, APOC4, DCD, LGALS3BP, FCN2, DOCK8, ANGPTL6, PRG4 | HPR, AGT, PRG4, HBA1, LPA, SLC4A1, SSC5D, PZP, APOD, APOE, APOC4 | PRG4, HPR, APOC4 | PRG4, APOC4 |
| **Accuracy (95% CI)** | 57.45 %  (46.82% - 67.59%) | 70.21%  (59.90 – 79.21%) | 58.51%  (47.88 – 68.59%) | 31.91%  (22.67 – 42.33%) | 73.40 %  (63.29 – 81.99%) |
| **No Information Rate (NIR)** | 72.34% | | | | |
| ***p*-value [ACC > NIR]** | 0.999 | 0.721 | 1.000 | 1.00 | 0.461 |
| **Kappa** | 0.28 | -0.04 | 0.04 | 0.02 | 0.46 |
| **McNemar’s Test p-value** | <0.001 | <0.001 | 0.337 | <0.001 | <0.001 |
| **Sensitivity** | 41.18% | 0.00% | 66.18% | 7.35% | 67.65% |
| **Specificity** | 100.00% | 97.06% | 38.46% | 96.15% | 88.46% |
| **Positive Predictive Value** | 100.00% | 0.00% | 73.77% | 83.33% | 93.88% |
| **Negative Predictive Value** | 39.39% | 71.74% | 30.30% | 28.41% | 51.11% |
| **Balanced Accuracy** | 70.59% | 48.53% | 52.32% | 51.75% | 78.05% |
| **Adjusted Rand Index** | -0.02 | -0.02 | 0.00 | 0.00 | 0.21 |

**Supplementary Table 3: Performance metrics of metabolomic signatures developed by different algorithms to differentiate plasma-derived EVs from patients with melanoma vs. healthy controls**

|  | **KNN Features** | **LASSO** | **Random Forest** | **Metabolites from all models** |
| --- | --- | --- | --- | --- |
| **Number of Metabolites** | 1 | 9 | 9 | 2 (LASSO & Random Forest) |
| **Metabolites** | PC ae C34:3 | PC ae C36:0, SM (OH) C22:2, lysoPC a C18:2, PC aa C36:2, PC aa C34:1, PC aa C30:0, PC aa C32:2, PC ae C34:3, PC ae C36:5 | PC ae C34:3, PC aa C40:5, SM C24:0, PC a C34:2, PC aa C40:4, PC aa C34:4, lysoPC a C18:2, PC ae C36:1, lysoPC a C16:0 | PC ae C34:3, lysoPC a C18:2 |
| **Accuracy (95% CI)** |  | 74.47 %  (59.65 – 86.06%) | 44.68%  (30.17 – 59.88%) | 82.98 %  (69.19 – 92.35%) |
| **No Information Rate (NIR)** |  | 72.34% | | |
| ***p*-value [ACC > NIR]** |  | 0.445 | 1.000 | 0.066 |
| **Kappa** |  | 0.42 | 0.12 | 0.59 |
| **McNemar’s Test p-value** |  | 0.387 | <0.001 | 0.723 |
| **Sensitivity** |  | 69.23% | 26.47% | 85.29% |
| **Specificity** |  | 76.47% | 92.31% | 76.92% |
| **Positive Predictive Value** |  | 52.94% | 90.00% | 90.62% |
| **Negative Predictive Value** |  | 86.67% | 32.43% | 66.67% |
| **Balanced Accuracy** |  | 72.85% | 59.39% | 81.11% |
| **Adjusted Rand Index** |  | 0.21 | -0.07 | 0.41 |

**Supplementary Table 4: Performance metrics of proteo-metabolomic signatures differentiating plasma-derived EVs of patients with melanoma vs. healthy controls**

|  | **Selected Features** | **Multi-algorithm  Proteo-metabolomic Signature** |
| --- | --- | --- |
| **Number of Features** | 4 | 2 |
| **Features** | PRG4, APOC4,  PC ae C34:3, lysoPC a C18:2 | PRG4, PC ae C34:4 |
| **Accuracy (95% CI)** | 82.98% (69.19 – 92.35%) | 85.11% (71.69 – 93.8%) |
| **No Information Rate (NIR)** | 72.34% | |
| ***p*-value [ACC > NIR]** | 0.066 | 0.031 |
| **Kappa** | 0.59 | 0.62 |
| **McNemar’s Test p-value** | 0.724 | 1.000 |
| **Sensitivity** | 85.29% | 91.18% |
| **Specificity** | 76.92% | 69.23% |
| **Positive Predictive Value** | 90.62% | 88.57% |
| **Negative Predictive Value** | 66.67% | 75.00% |
| **Balanced Accuracy** | 81.11% | 80.20% |
| **Adjusted Rand Index** | 0.41 | 0.46 |

**Supplementary Table 5: Performance metrics of proteomic signatures developed by different algorithms to differentiate plasma-derived EVs from patients with metastatic vs. primary melanoma**

|  | **KNN Features** | **LASSO** | **Random Forest** | **Proteins from all models** | **Multi-algorithm Protein Signature** |
| --- | --- | --- | --- | --- | --- |
| **Number of Proteins** | 10 | 9 proteins | 11 proteins | 6 proteins | 4 |
| **Proteins** | VWF, PLG, TNC, S100G, SERPIND1, APOC4, APOC3, C1R, ORM2, APOD | C1QB, PLG, TNC, S100G, F5, APOC4, SERPIND1, VWF, ACTB | VWF, TNC, S100G, HBD, PCY0X1, C4BPB, PLG, SERPIND1, APOL1, ORM2, APOC4 | TNC, PLG, VWF, S100G, SERPIND1, APOC4 | PLG, VWF, SERPIND1, TNC |
| **Accuracy (95% CI)** | 64.71%  (52.71 – 75.92%) | 51.47%  (39.03 – 63.78%) | 64.71%  (52.17. – 75.92%) | 50.00%  (37.62 – 62.38%) | 76.47%  (64.62 – 85.91%) |
| **No Information Rate (NIR)** | 64.71% | | | | |
| ***p*-value [ACC > NIR]** | 0.555 | 0.991 | 0.555 | 1.000 | 0.026 |
| **Kappa** | 0.23 | 0.15 | 0.28 | 0.05 | 0.51 |
| **McNemar’s Test p-value** | 1.000 | <0.001 | 0.153 | 0.010 | 0.211 |
| **Sensitivity** | 50.00% | 87.50% | 66.67% | 62.50% | 79.17% |
| **Specificity** | 72.73% | 31.82% | 63.64% | 43.18% | 75.00% |
| **Positive Predictive Value** | 50.00% | 41.18% | 50.00% | 37.50% | 63.33% |
| **Negative Predictive Value** | 72.73% | 82.35% | 77.78% | 67.86% | 86.84% |
| **Balanced Accuracy** | 61.36% | 59.66% | 65.15% | 52.84% | 77.08% |
| **Adjusted Rand Index** | 0.07 | -0.03 | 0.07 | -0.02 | 0.27 |

**Supplementary Table 6: Performance metrics of metabolomic signatures developed by different algorithms to differentiate plasma-derived EVs from patients with metastatic vs. primary melanoma**

|  | **KNN Model** | **LASSO** | **Random Forest** | **Metabolites from all models** | **Multi-algorithm Metabolite Signature** |
| --- | --- | --- | --- | --- | --- |
| **Number of Metabolites** | 5 | 1 | 9 | 3 *KNN & Random Forest* | 2 |
| **Metabolites** | PC ae C36:3, PC aa C34:3, PC aa C38:0, PC ae C40:1, PC ae C44:3 | PC ae C44:3 | SM C18:1, PC ae C36:4, PC ae C40:1. PC aa C38:0, lysoPC a C18:2, PC ae C44:3, Spermidine, SM C26:1, PC aa C36:0 | PC aa C38:0, PC ae C40:1, PC ae C44:3 | PC aa C38:0,  PC ae C44:3 |
| **Accuracy (95% CI)** | 44.12 %  (27.19 – 62.11%) |  | 67.67%  (49.47 – 82.61%) | 70.59%  (52.52 – 84.90%) | 70.59% (52.52 – 84.90%) |
| **No Information Rate (NIR)** | 64.71% |  | 64.71% | | |
| ***p*-value [ACC > NIR]** | 0.996 |  | 0.436 | 0.300 | 0.300 |
| **Kappa** | 0.11 |  | 0.18 | 0.24 | 0.24 |
| **McNemar’s Test p-value** | <0.001 |  | 0.076 | 0.027 | 0.027 |
| **Sensitivity** | 100.00% |  | 25.00% | 25.00% | 25.00% |
| **Specificity** | 13.64% |  | 90.91% | 95.46% | 95.46% |
| **Positive Predictive Value** | 38.71% |  | 60.00% | 75.00% | 75.00% |
| **Negative Predictive Value** | 100.00% |  | 68.97% | 70.00% | 70.00% |
| **Balanced Accuracy** | 56.82% |  | 57.96% | 60.23% | 60.23% |
| **Adjusted Rand Index** | -0.06 |  | 0.07 | 0.11 | 0.11 |

**Supplementary Table 7: Performance metrics of proteo-metabolomic signatures differentiating plasma-derived EVs of patients with metastatic vs. primary melanoma**

|  | **Selected Features** | **Multi-algorithm Proteo-metabolomic Signature** |
| --- | --- | --- |
| **Number of Features** | 6 | 2 |
| **Features** | PC ae C44:3, VWF, PLG, TNC, SERPIND1, PC aa C38:0 | VWF, SERPIND1 |
| **Accuracy (95% CI)** | 70.59% (52.52 – 84.90%) | 79.41% (62.10 – 91.30%) |
| **No Information Rate (NIR)** | 0.6471 | |
| ***p*-value [ACC > NIR]** | 0.300 | 0.049 |
| **Kappa** | 0.44 | 0.54 |
| **McNemar’s Test p-value** | 0.027 | 1.000 |
| **Sensitivity** | 91.67% | 66.67% |
| **Specificity** | 59.09% | 86.36% |
| **Positive Predictive Value** | 55.00% | 72.73% |
| **Negative Predictive Value** | 92.86% | 82.61% |
| **Balanced Accuracy** | 75.38% | 76.52% |
| **Adjusted Rand Index** | 0.14 | 0.32 |
